# Cognitive Flexibility Predicts Live-Fire Rifle Marksmanship in Airborne Cadets: A Pilot Study

**DOI:** 10.3390/brainsci15111150

**Published:** 2025-10-27

**Authors:** Dariusz Jamro, John A. Dewey, Grzegorz Żurek, Rui Lucena, Maciej Lachowicz

**Affiliations:** 1 Department of Physical Education and Sport, General Tadeusz Kosciuszko Military University of Land Forces, 51-147 Wroclaw, Poland; 2 Department of Psychological Science, University of North Georgia, Dahlonega, GA 30597, USA; 3 Department of Biostructure, Wroclaw University of Health and Sport Sciences, 51-612 Wroclaw, Poland; grzegorz.zurek@awf.wroc.pl (G.Ż.);; 4 Military Readiness Lab, 2720-113 Amadora, Portugal

**Keywords:** cognitive flexibility, inhibitory control, marksmanship, Army Combat Fitness Test, executive functions, human performance

## Abstract

Background: Executive functions may underpin performance in live-fire tasks, whereas evidence for global physical fitness is mixed. We quantified the associations between cognitive flexibility (CF), inhibitory control (IC), overall physical fitness, and rifle marksmanship in cadets, and derived a parsimonious predictive model. Methods: Twenty second-year male airborne cadets (mean age 21.7 ± 2.2 years) completed a live-fire Basic Rifle Marksmanship (BRM) qualification (40 targets at 50–300 m); the Color Trails Test (CTT-1 and CTT-2; interference index) to index CF and processing speed; a stop-signal–style task (CogniFit) to assess IC indexed by NO-GO accuracy and GO-trial response time; and the Army Combat Fitness Test (ACFT). Associations were examined with Spearman correlations. Multiple linear regression with backward elimination and Bayesian model comparison evaluated predictive models. Results: Faster CTT-2 performance was associated with higher BRM scores (ρ = −0.48, *p* = 0.032), with a similar association for CTT-1 (ρ = −0.46, *p* = 0.042). The best-fitting regression model included CTT-2 time and IC–accuracy (adjusted R^2^ = 0.345; RMSE = 7.03), with CTT-2 time the only significant predictor of BRM (b = −0.330, *p* = 0.006). Bayesian model comparison independently favored a parsimonious CTT-2–only model (P(M|data) = 0.222; BF_M_ = 5.41; BF_10_ = 1.00; R^2^ = 0.352). ACFT scores were not significantly associated with BRM. Conclusions: CF and processing speed are key correlates of live–fire rifle marksmanship in cadets, suggesting value in integrating executive–function elements into marksmanship training. Replication in larger cohorts is warranted.

## 1. Introduction

Combat readiness spans mobilization [1], technical/logistical [2], physical [3], and cognitive [4] domains, each essential for effective performance in complex military operations. Integrating these domains is linked to greater operational effectiveness and reduced risk under demanding conditions [1,3,4]. Shooting effectiveness (SE) is a core component of psychomotor readiness that influences lethality and small-unit success [5]. SE requires accurate fire, efficient weapon handling, rapid and correct decision-making, and sustained situational awareness.

Small-arms training systems continue to evolve with the dual goals of improving SE and training safety. Beyond basic marksmanship skills, instructors increasingly seek predictors of SE in psychological domains. The literature highlights candidates across cognitive (e.g., vigilance, spatial processing, cognitive flexibility (CF), inhibitory control (IC) [6]), affective (anxiety, psychophysiological states) [7], and psychomotor (grip, stance, heart rate, general fitness) [8] factors, with physical stressors further modulating performance [9].

Here we adopt the shorthand executive functions for core control processes supporting planning, execution, and monitoring of actions. Multiple studies indicate that higher executive functions levels, especially in CF and IC, are associated with superior SE, particularly in complex scenarios involving time pressure, unexpected targets, task switching, or elevated stress [7,10,11,12]. Under these conditions, attention control and inhibitory regulation enable faster, more accurate decisions, helping soldiers maintain accuracy and precision despite distraction and environmental volatility [7,9,12].

IC is a core executive function underlying accurate shoot/don’t-shoot decisions; stronger stopping ability reduces unintended casualties, and faster response time is associated with a higher number of lethal rounds, highlighting IC’s importance in high-risk contexts [13,14]. CF, the capacity to adapt strategies to novel or changing conditions, is also linked to superior SE in soldiers [10]. In complex engagements, CF supports rapid rule/task switching and efficient processing under pressure, sustaining accuracy and decision quality [15,16]. Executive function subcomponents such as selective attention, spatial orientation, and visual perception enable rapid target identification, prioritization of relevant cues, and suppression of distractors, thereby facilitating accurate fire [6]. Finally, by managing decision-making load under stress, greater CF is associated with better performance during armed confrontations and preserved accuracy despite cognitive demands [12,17].

Processing speed, an executive function facet, supports rapid multisensory integration and fine motor execution required for accurate fire. Neurophysiological evidence shows that skilled marksmen exhibit characteristic electrocortical patterns (e.g., increased alpha activity over left temporal regions) during shooting, consistent with more efficient information processing [18]. Under fire, decision-making is accompanied by neural signatures in regions subserving auditory and spatial processing, aiding localization and discrimination of critical cues [19]. Behaviorally, greater central visual processing capacity is associated with higher hit probability, underscoring the role of fast visual information processing in accuracy [20]. Longstanding work using response time as a proxy for processing speed in sport (including shooting) further supports these links [20]. Together, faster processing facilitates rapid threat discrimination and friend–foe differentiation, conserving time and cognitive resources during engagements.

Shooting experience is a key individual difference when predicting performance. Experts demonstrate stronger executive control than novices, suggesting that targeted training can enhance executive functions and, in turn, SE [21]; experimental work further indicates that cognitive training can reduce erroneous engagements in simulated environments [22].

Beyond executive functions, PF may also contribute to SE. Higher ACFT scores have been linked with better performance on selected shooting tasks [23]. Under operational constraints, movement capacity (e.g., agility and locomotor power) relates to tactical effectiveness under fire [24], and fatigue—particularly of the upper body—can degrade SE [25,26]. However, evidence on global PF and SE is mixed: in simulator-based work, PF predictors such as sprint speed and lower-body strength showed weak relationships with SE [24]. Instead, functional capacities that stabilize the firing platform (e.g., shoulder mobility and scapular control) appear more pertinent to accuracy, aligning with work on movement quality in tactical tasks and with observed links between higher ACFT scores and selected shooting outcomes [23,27].

Validated predictors and predictive models of SE can improve training efficiency and readiness. Identifying key cognitive and fitness determinants enables individualized practice and targeted remediation, while model-based screening helps allocate ammunition and time without compromising standards [5,28,29]. Accordingly, we aimed to determine the relationships between executive functions (IC and CF), overall PF, and basic rifle SE, and to develop a parsimonious predictive model of SE.

## 2. Materials and Methods

### 2.1. Participants

We conducted the study in a cohort of second-year airborne command-course cadets at the General Tadeusz Kościuszko Military University of Land Forces (Wrocław, Poland). The analytic sample comprised 20 male cadets (mean age 21.74 ± 2.15 years), all with comparable formal shooting experience (completion of the BRM course). All participants provided written informed consent and were medically cleared following history and physical examination. At the time of data collection, this specialty did not include female cadets.

The protocol was approved by the Research Ethics Committee of the Wrocław University of Health and Sport Sciences (Resolution No. 31/2025, approved on 17 July 2025) and complied with the Declaration of Helsinki and its amendments.

### 2.2. Method

#### Shooting Effectiveness

SE was indexed by scores from the BRM qualification. Testing was conducted on a standardized military range with moving and static 1:1 human silhouette targets presented at random intervals at 50–300 m. Cadets fired 40 rounds (5.56 mm) from M16A2 rifles with standard sight across four positions (prone unsupported, prone supported, kneeling supported, standing) per the course of fire; magazine changes followed the standard sequence (after the ninth round of each 10-shot string). The live-fire bout lasted approximately 4 min, and hits were recorded electronically. The outcome variable was a total BRM score (0–40, 1 point per hit).

### 2.3. Cognitive Flexibility

CF was assessed with the Color Trails Test (CTT), a culture-fair adaptation of the Trail Making Test that uses numbered, color-coded circles. Cadets completed CTT-1 (connecting numbers 1–25 in ascending order) followed by CTT-2 (connecting 1–25 while alternating colors) per standard instructions [30].

Primary outcomes were completion times for CTT-1 and CTT-2 (shorter times denote faster processing and better set-shifting). We also derived an interference index quantifying the additional control demands of CTT-2 relative to CTT-1:

$$\mathrm{Interference}\;\mathrm{index}=\frac{\mathrm{CTT}\text{-}2-\mathrm{CTT}\text{-}1}{\mathrm{CTT}\text{-}1}\;\times \;100\%$$

Higher interference index values indicate a greater set-shifting cost in CTT-2 relative to CTT-1, whereas smaller values reflect more efficient executive control and CF.

### 2.4. Inhibitory Control

**Figure 1:**
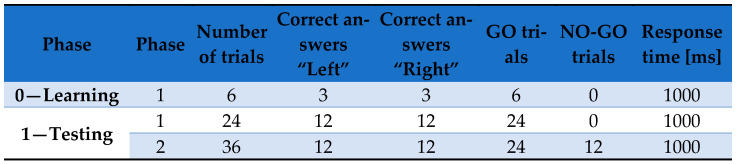
Protocol of presentation of GO and NO-GO stimuli in the stop-signal-style inhibition test (CogniFit). Source: own study based on: CogniFit Inc., San Francisco, CA, USA; https://www.cognifit.com/pl/battery-of-tests/focu-shif-test/inattention-test/ (accessed on 24 July 2025).

IC was assessed with a stop-signal-style inhibition task implemented in CogniFit; we reported IC indices (NO-GO accuracy; GO-trial response time) [31]. On each trial, one of two on-screen circles turned yellow (GO), to which participants responded as quickly as possible. On a subset of trials (12/36), the same circle subsequently turned red after a variable NO-GO signal delay (100, 200, or 300 ms; 4 trials each), instructing participants to withhold the response (Figure 1). GO and NO-GO trials were pseudorandomized. Primary outcomes were IC—accuracy (NO-GO accuracy) and IC—response time (GO response time); higher IC—accuracy indexes stronger IC, and shorter IC—response time indexes faster processing.

We did not estimate stop-signal reaction time (SSRT) because our field-compatible stop-signal-style inhibition task (Stop-Signal Test; CogniFit) did not implement the canonical staircase tracking of the stop-signal delay (SSD) that targets ~50% successful stopping and yields a broad range of SSDs, as recommended in the consensus guide [32]. The guide further recommends presenting stop signals on a minority of trials (≈25%) and including sufficient stop trials (≈≥50 for reliable group-level estimates); deviations from these design features, together with any violations of the independent race-model assumptions (e.g., when mean response time on unsuccessful stop trials is ≥mean go trial reaction time), can bias SSRT and should preclude its estimation. Accordingly, and in line with these recommendations, we treated accuracy as pragmatic outcome-based indices of response restraint, acknowledging that they are not substitutes for SSRT, which quantifies the latency of action cancelation. Future work would also implement staircase-based SSD and recommended SSRT estimation procedures (e.g., the integration method with replacement of go omissions) to assess whether action–cancelation latency adds predictive value for live-fire marksmanship beyond accuracy-based indices [32].

### 2.5. Physical Fitness

The Army Combat Fitness Test (ACFT) is a comprehensive PF test developed by the U.S. Armed Forces to assess soldiers’ fitness levels under realistic combat conditions. It was introduced to replace the previously used Army Physical Fitness Test (APFT) to better reflect the physical requirements of the modern battlefield and the diversity of military tasks [33].

Physical fitness was assessed using the six-event ACFT, administered in the standard order: 3-repetition maximum (3RM) deadlift; standing power throw with a 10-lb medicine ball; hand-release push-ups (2 min count); sprint–drag–carry (5 × 25 m shuttles: sprint, 180 lb sled drag, lateral shuffle, carry with two 40 lb kettlebells, sprint); plank hold; and 2 mile run. Outcome variables were the heaviest successful 3RM load, throw distance, number of correct push-ups in 2 min, sprint–drag–carry time, maximal plank time, and 2 mile time. A standardized 15 min warm-up preceded testing; rest intervals were 2 min between events 1–5 and 10 min before the run. Higher event performance yields a higher ACFT score.

### 2.6. Statistical Analysis

We summarized all variables with descriptive statistics (mean, SD, 95% CI, range, coefficient of variation). Distributions were inspected (Shapiro–Wilk) to guide choice of correlation: Pearson for approximately normal variables and Spearman otherwise (two-tailed, α = 0.05).

To model SE (BRM), we first examined scatterplots and multicollinearity (variance inflation factors, VIF). Because CTT-1, CTT-2, and the interference index were highly collinear (VIF > 10), we retained CTT-2 and excluded the other two from regression. We then fit multiple linear regressions with BRM as the dependent variable and candidate predictors CTT-2, IC—response time, IC—accuracy, and ACFT, applying backward elimination (remove *p* > 0.05) to obtain a parsimonious model. Model performance was evaluated using R^2^, adjusted R^2^, R^2^ change, F change, and RMSE; ANOVA provided global tests. Assumptions were checked via residual diagnostics (normality: Shapiro–Wilk and Q–Q plots; homoskedasticity: Breusch–Pagan; independence: Durbin–Watson).

In addition to the frequentist analyses, we conducted Bayesian Linear Regression for BRM. We evaluated all sensible model combinations comprising CTT-2, IC—response time, IC—accuracy, and ACFT, alongside the null model. We used JASP’s default priors (JZS g-prior for regression coefficients and a noninformative prior for the error variance; default model priors as implemented in JASP). Model comparison was based on Bayes factors versus the null (BF_10_), posterior model probabilities P(M|data), and BFM (posterior-to-prior odds). For each predictor, we report the Bayes factor for inclusion (BF_incl_) and the posterior inclusion probability P(incl|data). Posterior summaries are presented as posterior means, SDs, and 95% credible intervals; we also report descriptive R^2^ for model fit.

Analyses were conducted in IBM SPSS Statistics 27 and JASP 0.19.2.

## 3. Results

Table 1 summarizes all variables. BRM scores averaged 28.85 ± 8.69 (range 14–39). CTT-2 times averaged as 65.15 ± 15.51 s (range 52 s). ACFT totaled 546.7 ± 36.53 points (range 120). The interference index averaged as 127% ± 35.58%. The largest variability was observed for CTT-2 and BRM.

As shown in Table 2, BRM correlated negatively with CTT-1 (r = −0.458, *p* = 0.042) and CTT-2 (r = −0.481, *p* = 0.032). CTT-2 correlated positively with the interference index (r = 0.508, *p* = 0.022). IC—response time correlated strongly with IC—accuracy (r = 0.756, *p* < 0.001). Neither IC metric nor ACFT correlated significantly with BRM.

We fitted multiple linear regressions with backward elimination. Across all specifications, CTT-2 time is the only stable, statistically significant predictor of BRM score (b range = −0.310 to −0.332; β = −0.554 to −0.593; t = −2.61 to −3.17; *p* = 0.016–0.006). Faster CTT-2 performance (shorter time) is associated with higher marksmanship. IC—accuracy shows a positive but nonsignificant association (e.g., in M_2_: b = 0.480, β = 0.250, *p* = 0.196). IC—response time (b ≈ −0.021, *p* ≥ 0.668) and ACFT score (b = −0.013, *p* = 0.789) are not significant (Table 3). 

Reduced models are significant overall: M_1_, F(3,16) = 3.877, *p* = 0.029; M_2_, F(2,17) = 6.006, *p* = 0.011; M_3_, F(1,18) = 9.765, *p* = 0.006. The full model M_0_ does not reach conventional significance, F(4,15) = 2.758, *p* = 0.067 (Table 4).

The best-fitting model is M_2_ (CTT-2 + IC—accuracy) with Adjusted R^2^ = 0.345 and RMSE = 7.032 (R = 0.643). The parsimonious M_3_ (CTT-2 only) remains significant with slightly lower fit (Adjusted R^2^ = 0.316; RMSE = 7.188). M_1_ shows similar fit (Adjusted R^2^ = 0.312; RMSE = 7.205), whereas M_0_ performs worst (Adjusted R^2^ = 0.270; RMSE = 7.423) (Table 5).

CTT-2 time is a robust, independent predictor of marksmanship. While IC—accuracy does not attain individual significance, adding it to CTT-2 yields a small improvement in model fit, suggesting modest incremental explanatory value.

The posterior model probabilities favored the CTT-2-only specification (P(M|data) = 0.222; BFM = 5.41; BF_10_=1.00; R^2^ = 0.352). Adding additional predictors did not increase support despite slightly larger R^2^ values: the full model with CTT-2 + IC—response time + IC—accuracy + ACFT showed P(M|data) = 0.157; BFM = 0.75; BF_10_ = 0.177; and R^2^ = 0.424, and CTT-2 + IC—accuracy yielded P(M|data) = 0.108; BFM = 3.53; BF_10_ = 0.734; and R^2^ = 0.414. Models without CTT-2 received very low support (e.g., IC—accuracy alone: P(M|data) = 0.018; BF_10_ = 0.082; R^2^ = 0.068, and ACFT alone: P(M|data) = 0.012; BF_10_ = 0.056; R^2^ = 0.013), indicating little to no evidence relative to the null. Overall, the evidence favors a parsimonious model centered on CTT-2, with only anecdotal gains from adding other predictors (Table 6).

The posterior inclusion probability (PIP) was highest for CTT-2 (P(incl|data) = 0.811; BF_incl_ = 4.297), constituting moderate evidence for inclusion. Its posterior mean effect was −0.201 (SD = 0.132) with a 95% credible interval [−0.416, 0.000], consistent with a negative association (shorter time—higher BRM score). All other predictors had PIP < 0.50 and BF_incl_ < 1 (IC—response time: PI*P* = 0.388, BF_incl_ = 0.634; IC—accuracy: PI*P* = 0.461, BF_incl_ = 0.855; ACFT: PI*P* = 0.368, BF_incl_ = 0.583), and their credible intervals broadly spanned zero, providing evidence against their inclusion (Table 7).

Bayesian model evidence converges with the frequentist results: CTT-2 time is the sole robust predictor of marksmanship. Adding IC—accuracy or other variables yields, at best, a limited incremental value that is not supported once model complexity is penalized.

## 4. Discussion

The present study evaluated whether executive functions, CF and IC, and overall PF explain variance in rifle SE during standardized BRM qualification. Across analyses, faster CTT-2 performance was associated with higher BRM and remained the only significant predictor after model selection; Bayesian model comparison likewise favored a parsimonious CTT-2-only specification and provided moderate evidence for including CTT-2 (Table 3, Table 4, Table 5, Table 6 and Table 7). Adding IC—accuracy produced a small descriptive gain in the frequentist models (adjusted R^2^ = 0.345; RMSE = 7.03 vs. 7.19), but the coefficient did not reach significance and Bayesian evidence for its inclusion was against, indicating that IC’s incremental contribution is tentative in this dataset. Global PF (ACFT total) showed no association with BRM. Taken together, these findings suggest that flexibility under alternating rules and speeded processing—as indexed by CTT-2—are more proximal determinants of BRM performance than broad fitness measures; any added value of IC should be verified in larger samples.

Our correlation and modeling results align with the expanding literature demonstrating that executive functions contribute to SE above and beyond basic technical skills and tactics. Studies in cadets and soldiers report positive links between executive functions and SE in tasks that emulate operational complexity, time pressure, and distraction [6,7,8,9,10,12]. In our data, CTT-2 (requiring rapid set-shifting with color alternation) explained the unique variance in BRM, consistent with evidence that flexibility supports the switching of visuospatial rules, priority maps, and response strategies when the tactical context changes [10]. This pattern also coheres with research showing that performance in complex, dynamic engagements depends on the ability to reconfigure attention and control prepotent but inappropriate responses under pressure [7,12]. By design, CTT-2 taxes precisely those capacities, alternation, interference control, and efficient allocation of attention that are likely recruited when shooters update their posture, sight alignment, and shoot/don’t shoot decisions across quickly changing target presentations.

On the battlefield, shooters must frequently withhold fire in the presence of friendly forces or noncombatants (e.g., room clearing in urban environments or counterterrorism operations). In such contexts, performance depends not only on rapid target detection but on context-contingent suppression of prepotent firing responses; conceptual and experimental work links inhibitory control to shoot/don’t-shoot accuracy and reductions in erroneous fire under risk [13,15], cognitive training can reduce unintended engagements [22], and friend–foe identification and decision accuracy degrade under heavy cognitive load and time pressure [34]. In our study, IC was operationalized as NO-GO/stop-trial accuracy together with GO-trial response time; we did not estimate Stop Signal Reaction Time (SSRT), which quantifies the covert latency of action cancelation and requires staircase tracking of the stop-signal delay targeting ~50% successful stops, as recommended in the consensus guide [32]. Because the BRM administered here imposed relatively few explicit no-shoot demands, this operationalization likely underweighted inhibition requirements and may help explain why the IC—accuracy signal was only tentatively relative to CTT-2. Future work should embed frequent no-shoot cues and ambiguous friend–foe discriminations and estimate SSRT alongside accuracy to capture inhibition capacity more comprehensively [32].

A convergent line of evidence indicates that faster information processing supports marksmanship. Classic psychophysiological work shows that skilled shooters exhibit distinct electrocortical profiles during performance, interpreted as signatures of efficient sensory-motor integration [18]. Under fire, decision processes engage neural systems for auditory and spatial cue integration that support rapid localization and discrimination of task-relevant signals [19]. At the behavioral level, greater central visual processing capacity predicts higher hit probability in simulated fire team engagements, and response time has long served as a proxy for processing speed in sport, including shooting [34]. Our finding that shorter CTT-2 times (which reflect both set-shifting and processing speed) predicted BRM is therefore consistent with neurobehavioral accounts: faster uptake and updating of visual information should facilitate quicker micro-adjustments of posture, sight picture, and trigger control, yielding higher hit counts when target windows are brief.

Prior work suggests that experienced shooters show stronger cognitive control than novices, and that training can improve executive functions with downstream benefits to SE [21]. While our cohort was relatively homogeneous in formal experience (completed the BRM course), the observed variance in CTT-2 and its association with BRM is consistent with the view that individual differences in executive functions, shaped by experience and trainability, contribute meaningfully to live-fire outcomes. This interpretation is further supported by demonstrations that targeted cognitive training reduces erroneous shootings in simulated conditions [22]. Longitudinal designs will be required to confirm whether executive function gains translate into durable improvements in BRM and whether those gains generalize to more complex, collective tasks.

The lack of association between ACFT and BRM in our sample echoes reports that global fitness predictors (e.g., speed, lower-body strength) show weak or non-significant links to SE in simulator-based assessments [24]. At the same time, there is evidence that selected elements of fitness align with task performance in shooting-relevant contexts; for example, higher ACFT performance has been related to better outcomes in specific marksmanship tasks [23], and movement quality constructs that stabilize the firing platform (e.g., shoulder mobility, scapular control) have been connected to tactical performance [27]. These findings point to role specificity: physical attributes that stabilize the weapon and sustain fine motor control under load may matter more for accuracy than broad fitness composites. Moreover, acute psychophysiological stressors and fatigue, especially of the upper body, can impair shooting behavior and SE in duty-relevant settings [9,25,26]. The present results therefore should not be taken to downplay the importance of physical preparation overall, but rather to emphasize that, for BRM-style accuracy outcomes, CF and processing speed may explain more variance than global fitness scores in relatively rested conditions.

Decision-making in armed confrontation requires rapid prioritization of cues, suppression of inappropriate responses, and flexible shifting between rules under uncertainty processes that the executive function framework captures [15,16]. Our pattern of finding significant CTT-2 effects and incremental values of an IC index fit this mechanistic picture. Under alternating rules and time limits, shooters with better set-shifting and quicker updating likely form and refresh action plans more efficiently, maintain a stable sight picture under distraction, and correct errors sooner. When go/stop contingencies become frequent or ambiguous, inhibitory demands increase, and IC may become a stronger determinant of outcomes [13,14,34]. Neurophysiological observations of skilled marksmen characteristic alpha activity and efficient engagement of sensory areas [18,19] are consistent with such efficient control loops operating at pace. From a training perspective, this implies that optimizing executive function capacities relevant to the engagement type (set-shifting, stopping, visual pickup) should yield the greatest marginal benefit.

The finding that CF and processing speed predicts live-fire qualification, whereas broad fitness does not, suggests task-congruent interventions. Combining live-fire or dry-fire drills with CF challenges rapid rule alternation, target-feature switching, and go/stop discrimination may reinforce shoot/don’t-shoot control and attentional switching [7,12,13,14,22]. Visual-processing work that improves central cue uptake (e.g., drills for rapid acquisition and discrimination) could further increase hit probability under time pressure [34]. On the physical side, training should emphasize functional capacities that stabilize the firing platform (shoulder/scapular control, postural endurance) rather than relying on global conditioning alone, consistent with mixed PF findings and links to task outcomes [23,24,27]. Finally, fatigue management is essential: upper-body and whole-body fatigue can degrade accuracy in contexts analogous to duty demands [25,26]. These implications are actionable within existing training time by embedding brief executive function elements into standard marksmanship progressions and by prioritizing shoulder girdle and postural stability within strength programs.

Our results extend observations in cadets that executive function metrics correlate with SE by showing that a simple, widely used CF index (CTT-2 time) robustly predicts live-fire BRM scores. In contrast to research that emphasizes IC in shoot/don’t-shoot paradigms [13,14,22,35], our BRM task places relatively fewer explicit stop demands; accordingly, IC—accuracy provided at most a tentative, small, incremental signal and contributed less strongly than CF. We also clarify the PF relation by showing no association between ACFT total and BRM, complementing mixed findings from simulator-based and task-specific studies [24,35]. Taken together, these findings support a parsimonious emphasis on CF, particularly flexible, speeded processing indexed by CTT-2—while treating IC as context-dependent: its added value is more likely when engagement conditions embed strong go/stop contingencies or rapid rule reversals. Practically, the results support integrating executive function-targeted micro-drills (rule shifts/switching, speeded processing, and, where relevant, inhibition) into weapons training to enhance SE.

### Limitations

Despite the valuable findings, this study has limitations that could be taken into account. The study group consisted of second-year cadets with limited shooting experience, which may affect the extent of generalization in relation to more experienced soldiers. The modest sample size (N = 20) constrained statistical power, particularly for detecting small-to-moderate effects: some non-significant associations observed here may have reached significance in a larger cohort. Nevertheless, our inferences emphasize the largest effects which are most consequential from a practical standpoint and should be corroborated in adequately powered, multi-cohort studies.

Furthermore, the study focused on assessing SE solely in the context of qualification shooting in BRM under controlled conditions. Although the shooting task was dynamic, including stance and magazine changes, the direct impact of stressors or overload factors such as physical fatigue, combat stress, or multitasking, which can be important in real-world operational conditions, was not investigated.

Ultimately, executive functions represent a complex set of cognitive processes that involve different components which cannot be captured by single cognitive tests. The present study focused on assessing selected executive function aspects, such as CF, speed of information processing, and IC with the use of CTT and stop-signal-style inhibition tasks (CogniFit). Therefore, the results should not be directly generalized to all components of executive functions. Future research could take into account a wider range of executive function tests, such as tests of working memory, visuospatial processing, or decision-making under time pressure, which would allow a more comprehensive analysis of the role of cognitive functions in military marksmanship and their impact on performance under stress.

## 5. Conclusions

The findings of the present study highlight the key role of CF and speed of information processing in SE, especially under dynamic combat conditions. The CTT-2 completion time was found to be the strongest BRM performance predictor, which indicates the importance of CF and the ability to switch attention quickly, analyze visuospatial stimuli, and make decisions under time pressure for SE. The absence of significant correlations between overall PF level and BRM performance suggests that motor skills, albeit of crucial importance in soldiers’ overall combat preparation, are not a direct determinant of SE.

The practical implications of the studies suggest a proposal to expand training programs and aptitude verification among soldiers by adding elements of executive function level testing. As it appears, selected executive functions can support soldiers’ ability to effectively acquire targets, perform rapid situational analysis and optimize responses in dynamic combat environments. The study provided new insights into the synergy of cognitive and physical abilities in specific military skills, enabling better adjustment of training processes to maximize SE.

## Figures and Tables

**Table 1:** Descriptive statistics.

		95% CI Mean									
	Mean	Uper	Lower	SD	CV	Variance	Shapiro–Wilk	*p*-Value of Shapiro–Wilk	Min.	Max.	
CTT-1 [s]	29.30	32.00	26.60	5.77	0.20	33.27	0.97	0.76	18.00	39.00	
CTT-2 [s]	66.15	73.41	58.89	15.51	0.23	240.56	0.94	0.25	43.00	95.00	
Interference index [%]	127.00	143.65	110.35	35.58	0.28	1265.89	0.93	0.19	73.00	183.00	
IC—response time [ms]	485.95	511.99	459.91	55.64	0.11	3095.63	0.97	0.73	372.00	591.00	
IC—accuracy [%]	92.05	94.17	89.93	4.52	0.05	20.47	0.98	0.94	83.00	100.00	
BRM [score]	28.85	32.92	24.78	8.69	0.30	75.50	0.89	0.03	14.00	39.00	
ACFT [score]	546.70	563.80	529.60	36.53	0.07	1334.43	0.90	0.05	470.00	590.00	

Abbreviations: CI, Confidence interval; SD, Std. deviation; CV, Coefficient of variation; CTT, Color Trails Test; IC, Inhibitory control; BRM, Basic Rifle Marksmanship; ACFT, Army Combat Fitness Test.

**Table 2:** The results of the correlation analysis between the analyzed variables.

		*r*	ρ	*p*-Value	Effect size (Fisher’s z)	Std. Error (z)
CTT-1 [s]	CTT-2 [s]	0.736 **		<0.001	0.942	0.243
CTT-1 [s]	Interference index [%]	−0.196		0.407	−0.199	0.243
CTT-1 [s]	IC—response time [ms]	0.211		0.371	0.215	0.243
CTT-1 [s]	IC—accuracy [%]	0.030		0.901	0.030	0.243
CTT-1 [s]	BRM [score]		−0.458 *	0.042	−0.494	0.244
CTT-1 [s]	ACFT [score]		−0.104	0.661	−0.105	0.237
CTT-2 [s]	Interference index [%]	0.508 *		0.022	0.561	0.243
CTT-2 [s]	IC—response time [ms]	0.237		0.315	0.241	0.243
CTT-2 [s]	IC—accuracy [%]	−0.020		0.935	−0.020	0.243
CTT-2 [s]	BRM [score]		−0.481 *	0.032	−0.525	0.244
CTT-2 [s]	ACFT [score]		−0.094	0.694	−0.094	0.237
Interference index [%]	IC—response time [ms]	0.081		0.735	0.081	0.243
Interference index [%]	IC—accuracy [%]	−0.050		0.833	−0.050	0.243
Interference index [%]	BRM [score]		−0.179	0.450	−0.181	0.239
Interference index [%]	ACFT [score]		−0.078	0.745	−0.078	0.237
IC—response time [ms]	IC—accuracy [%]	0.756 **		<0.001	0.986	0.243
IC—response time [ms]	BRM [score]		0.075	0.753	0.075	0.237
IC—response time [ms]	ACFT [score]		0.142	0.550	0.143	0.238
IC—accuracy [%]	BRM [score]		0.274	0.242	0.281	0.240
IC—accuracy [%]	ACFT [score]		0.114	0.633	0.114	0.238
BRM [score]	ACFT [score]		0.088	0.712	0.088	0.237

Statistically significant correlations are marked in bold; * *p* < 0.05, ** *p* < 0.001. Abbreviations: CTT, Color Trails Test; IC, Inhibitory control; BRM, Basic Rifle Marksmanship; ACFT, Army Combat Fitness Test.

**Table 3:** Coefficients.

Model		*b*	Std. Error	β (Standardized)	t	*p*
M_0_	(Intercept)	3.524	47.907		0.074	0.942
	CTT-2 [s]	−0.317	0.122	−0.566	−2.606	0.020
	IC—response time [ms]	−0.021	0.051	−0.137	−0.422	0.679
	IC—accuracy [%]	0.694	0.607	0.361	1.143	0.271
	ACFT [score]	−0.013	0.048	−0.055	−0.273	0.789
M_1_	(Intercept)	−2.832	40.618		−0.070	0.945
	CTT-2 [s]	**−0.310**	0.115	−0.554	−2.686	0.016
	IC—response time [ms]	−0.021	0.049	−0.137	−0.437	0.668
	IC—accuracy [%]	0.680	0.587	0.354	1.158	0.264
M_2_	(Intercept)	6.501	33.709		0.193	0.849
	CTT-2 [s]	**−0.330**	0.104	−0.588	−3.167	0.006
	IC—accuracy [%]	0.480	0.357	0.250	1.345	0.196
M_3_	(Intercept)	50.828	7.215		7.045	<0.001
	CTT-2 [s]	**−0.332**	0.106	−0.593	−3.125	0.006

Statistically significant variables are marked in bold. Abbreviations: M, Model; CTT, Color Trails Test; IC, Inhibitory control; BRM, Basic Rifle Marksmanship; ACFT, Army Combat Fitness Test.

**Table 4:** ANOVA.

Model		Sum of Squares	df	Mean Square	F	*p*
M_0_	Regression	607.951	4	151.988	2.758	0.067
	Residual	826.599	15	55.107		
	Total	1434.550	19			
M_1_	Regression	603.858	3	201.286	3.877	0.029
	Residual	830.692	16	51.918		
	Total	1434.550	19			
M_2_	Regression	593.957	2	296.978	6.006	0.011
	Residual	840.593	17	49.447		
	Total	1434.550	19			
M_3_	Regression	504.533	1	504.533	9.765	0.006
	Residual	930.017	18	51.668		
	Total	1434.550	19			

Abbreviations: M, Model; F, Fisher’s F statistic; df, Degrees of freedom.

**Table 5:** Model summary—BRM [score].

Model	R	R^2^	Adjusted R^2^	RMSE	R^2^ Change	F Change	df1	df2	*p*
M_0_	0.651	0.424	0.270	7.423	0.424	2.758	4	15	0.067
M_1_	0.649	0.421	0.312	7.205	−0.003	−0.079	1	16	1.000
M_2_	0.643	0.414	0.345	7.032	−0.007	−0.200	1	17	1.000
M_3_	0.593	0.352	0.316	7.188	−0.062	−1.731	1	18	1.000

Abbreviations: R^2^, Coefficient of determination; RMSE, Root mean square error; df, Degrees of freedom.

**Table 6:** Model comparison—BRM [score].

Models	P(M)	P(M|Data)	BF_M_	BF_10_	R^2^
CTT-2 [s]	0.050	0.222	5.411	1.000	0.352
CTT-2 [s] + IC—response time [ms] + IC—accuracy [%] + ACFT [score]	0.200	0.157	0.746	0.177	0.424
Null model	0.200	0.115	0.521	0.130	0.000
CTT-2 [s] + IC—accuracy [%]	0.033	0.108	3.528	0.734	0.414
CTT-2 [s] + IC—response time [ms] + IC—accuracy [%]	0.050	0.076	1.572	0.345	0.421
CTT-2 [s] + IC—accuracy [%] + ACFT [score]	0.050	0.073	1.504	0.331	0.417
CTT-2 [s] + IC—response time [ms]	0.033	0.069	2.160	0.469	0.372
CTT-2 [s] + ACFT [score]	0.033	0.056	1.735	0.382	0.352
CTT-2 [s] + IC—response time [ms] + ACFT [score]	0.050	0.048	0.964	0.218	0.374
IC—accuracy [%]	0.050	0.018	0.350	0.082	0.068
ACFT [score]	0.050	0.012	0.240	0.056	0.013
IC—response time [ms]	0.050	0.011	0.220	0.052	0.000
IC—response time [ms] + IC—accuracy [%]	0.033	0.011	0.331	0.076	0.160
IC—response time [ms] + IC—accuracy [%] + ACFT [score]	0.050	0.010	0.183	0.043	0.163
IC—accuracy [%] + ACFT [score]	0.033	0.006	0.186	0.043	0.074
IC—response time [ms] + ACFT [score]	0.033	0.004	0.128	0.030	0.013

Abbreviations: BRM, Basic Rifle Marksmanship; CTT-2, Color Trails Test-Part 2; IC, Inhibitory control; ACFT, Army Combat Fitness Test; BF_10_, Bayes factor vs. null; BF_m_, Posterior-to-prior odds ratio; P(M), Prior model probability; P(M|data), posterior model probability.

**Table 7:** Posterior summaries of coefficients.

								95% CI	
Coefficient	P(Incl)	P(Excl)	P(Incl|Data)	P(Excl|Data)	BF_incl_	Mean	SD	Lower	Upper
Intercept	1.000	0.000	1.000	0.000	1.000	28.850	1.689	25.517	32.371
CTT-2 [s]	0.500	0.500	0.811	0.189	4.297	−0.201	0.132	−0.416	0.000
IC—response time [ms]	0.500	0.500	0.388	0.612	0.634	−0.002	0.026	−0.083	0.051
IC—accuracy [%]	0.500	0.500	0.461	0.539	0.855	0.201	0.369	−0.366	1.142
ACFT [score]	0.500	0.500	0.368	0.632	0.583	−0.002	0.025	−0.074	0.056

Abbreviations: BRM, Basic Rifle Marksmanship; CTT-2, Color Trails Test; IC, Inhibitory control; ACFT, Army Combat Fitness Test; P(incl), Prior inclusion probability of a predictor; P(excl), Prior exclusion probability; P(incl|data), Posterior inclusion probability (PIP); P(excl|data), Posterior exclusion probability; BF_incl_, Bayes factor for inclusion; SD, Posterior standard deviation; CI, Credible interval.

## Data Availability

The raw data presented in the study are not publicly available due to the need to protect participants’ privacy under the European General Data Protection Regulation and in accordance with internal military regulations governing the handling of data on active-duty service members and cadets (operational security and confidentiality). To request access, please contact the first author; any sharing of de-identified data will be subject to institutional and military approvals.
